# Recent Advances and Challenges in Cancer Care, with a Focus on Multiple Myeloma, Lymphoma, and Lung Cancer: Key Insights from the Onco Summit 2024-The APAC Chapter

**DOI:** 10.31557/APJCP.2025.26.5.1543

**Published:** 2025

**Authors:** Ja Min Byun, Won Seog Kim, Myung-Ju Ahn

**Affiliations:** 1 *Division of Hematology and Oncology, Department of Internal Medicine, Seoul National University Hospital, Seoul, Korea. *; 2 *CAR-T Cell Therapy Center & Division of Hematology-Oncology, Samsung Medical Center, Sungkyunkwan University School of Medicine, Seoul, Korea. *; 3 *Division of Hematology-Oncology, Department of Medicine, Samsung Medical Center, Sungkyunkwan University School of Medicine, South Korea. *

**Keywords:** Cancer care challenge, Diagnostic advances, Therapeutic advances, Lung cancer, Multiple myeloma

## Abstract

The Onco Summit 2024: The APAC Chapter took place over 2 days, between 16th and 17th February 2024, in South Korea. The event aimed to provide healthcare professionals with the latest updates in different oncology disciplines and share best practices and strategies to ensure optimal patient care. The Summit aimed to foster international and local collaborations to address challenges, understand the future of oncology, and explore opportunities to improve cancer care, especially in the Asia Pacific region. There were 27 scientific presentations and 10 panel discussions with engaging discussions around recent diagnostic and therapeutic advances in cancer care and associated challenges. More than 150 oncology specialists from 11 countries attended the Summit. The Summit highlighted specific challenges in the Asia Pacific region and discussed transferrable strategies to address some of these challenges. This report aims to present a summary of key discussions from the Summit for the global and Asia Pacific oncology community.

## Introduction

The Asia Pacific (APAC) region represents half of the cancer cases worldwide and 58% of cancer-related deaths [1]. In 2019, one-fourth of the deaths from non-communicable diseases were due to cancer in this region, making it the most heavily burdened by cancer [1]. This is further exacerbated by the varying income levels across Asian countries [1-3]. Although there is a high incidence of cancer in higher-income countries, the mortality rates are significantly higher in low- and middle-income countries, thereby highlighting the disparity in healthcare systems and financial support from the government [1-5]. 

Each country in APAC presents unique characteristics, cultures, and linguistics that thereby influence the incidence, risk factors, needs, and challenges in providing cancer care [1-5]. Health inequalities have emerged as a significant challenge in this region with accessibility to screening, molecular testing, and novel therapies remaining limited across majority of the Asian population [6]. Although cancer screening and early detection have proven to reduce cancer burden, national screening programs for cancer are either not well established in the APAC region or have other challenges such as limited participation, financial constraints, limited access, or cultural restraints [5]. Besides cultural, sociodemographic, geographic, religious, and ethnic challenges, scarce healthcare facilities, out-of-pocket expenses, lack of national registries, and suboptimal supportive care further complicate cancer care in this region [5, 7]. Despite the recent advances in diagnostics and therapeutics, disparities remain in the needs and capabilities within the APAC region [5]. The use of novel diagnostic and therapeutic interventions requires a thorough understanding of their role and action, staying updated on the constantly evolving diagnostic and therapeutic landscape, understanding optimal treatment choice and sequencing tailored to patient needs, and optimal implementation in clinical settings. 

**Figure 1:**
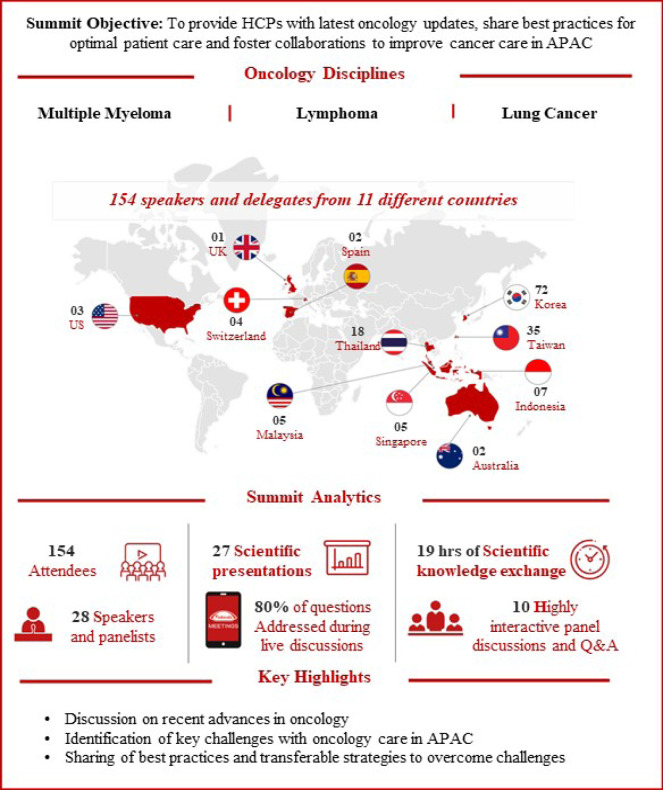
APAC Oncology Summit Overview. APAC: Asia Pacific; HCP: healthcare professional; Q&A: questions and answers.

Educational and awareness programs are important for addressing the challenges associated with cancer burden in the APAC region. The Onco Summit 2024: The APAC Chapter was an educational initiative sponsored by Takeda and held in South Korea to discuss some of the global and APAC-specific challenges associated with cancer care and share best practices for addressing these challenges. This report presents a summary of the key discussions (Figure 1) about the most recent developments and challenges in oncology clinical practice and oncology research from the Summit.

### Overview of the APAC ONCO SUMMIT 2024

The 2024 Onco Summit: APAC Chapter was a Takeda-sponsored educational event held in Seoul, Republic of Korea, on 16th and 17th February 2024. The aims of the summit were to provide healthcare professionals in APAC with unbiased, scientific updates in different oncology fields and provide a collaborative platform to share insights and strategies to address challenges and understand the future of oncology. The Summit also aimed to foster international and local collaborations to work together to ensure the best treatment for all patients. Key discussions were held on the topics of multiple myeloma, Hodgkin lymphoma, and non-small cell lung cancer (NSCLC). Over 150 attendees from 11 countries engaged in 27 scientific presentations and 10 interactive panel discussions (Figure 1).

### Recent advances in oncology

*Multiple myeloma*

Tailoring therapeutic strategies in multiple myeloma

Owing to the many different clinical variables in patients with newly diagnosed multiple myeloma (NDMM), such as sex, age, and chromosomal abnormalities, clinical outcomes in these patients are heterogeneous. While enhancing overall survival is key, it is important to understand patient needs, the available drugs, and the local and regulatory circumstances. Patients should be informed that the goal of multiple myeloma treatment is not to cure, but to relieve symptoms, for which several options are available. With the growing number of treatment options available, it is necessary to understand the mechanism of actions, adverse events, efficacy, and combination strategies for different available treatments. Additionally, it is essential to consider what matters the most to patients and patient perspectives to improve multiple myeloma (MM) care and quality of life. Data from real-world studies can guide treatment decisions in myeloma as well as be used to inform patients about drugs, treatment options, and goals of the treatment to trigger confidence in patients regarding treatment options. Furthermore, local access and reimbursement policies within a country, local clinical practice settings, local health policies and guidelines, and local regulatory settings, tend to influence treatment choices in multiple myeloma. Real-world evidence should guide changes in treatment guidelines and clinical practice to best support clinician decisions. 

To personalize therapy, stratification needs to be performed based on tumor biology (genomic changes in plasma), tumor burden, and the host biology (fitness vs frailty). Therapy can be tailored at baseline and after initial set of treatment. In addition to patient-, drug-, and local regulatory-related factors, several clinical factors are considered while tailoring treatment at baseline, such as transplant eligibility, frailty of transplant-ineligible patients, and genomic make-up of myeloma. Genomic changes that are used to stratify patients include cytogenic abnormalities having a prognostic significance [8]. High-risk patients can be identified using several approaches such as R-ISS, fluorescent in-situ hybridization (FISH), and SKY92. While tailoring therapy after initial treatment, certain factors such as tumor burden post-therapy and patient frailty need to be considered. Minimal residual disease (MRD) negativity is associated with better progression-free survival (PFS) outcomes, as demonstrated by an expanded meta-analysis [9]. Frailty of a patient decreases from baseline to year 3, which may be attributed to improved outcomes after treatment [10]. Therapy can be tailored in three ways—differentiation, escalation, or intensification and de-escalation or discontinuation of therapy. Escalation of therapy can be performed through tandem transplants and augmented consolidation and maintenance. De-escalation of therapy can be performed through fixed-duration therapy and by determining therapy duration by response. Completed (STAMINA, EMORY 1000, PERSEUS, FORTE, and EMNO2 trials) and ongoing (FITness and RADAR trials) clinical trials can support and guide stratification and tailoring decisions [11-14]. 

### Salvage therapies for lenalidomide- or daratumumab-exposed patients

Several challenges are faced while treating relapsed refractory multiple myeloma (RRMM). One significant issue is the genomic complexity, particularly clonal evolution [15-17]. After undergoing multiple lines of therapy, patients show variability at relapse with respect to prior treatments, their comorbidities, and refractoriness to specific therapies [18]. For patients with RRMM progressing on lenalidomide and/or daratumumab, treatment needs to be tailored. 

To identify lenalidomide refractory patients, it is important to consider the dose of lenalidomide used, duration of treatment, and patient compliance to treatment as poor compliance is common among elderly patients who are either frail or on multiple treatments [19, 20]. In Korea, most patients newly diagnosed with multiple myeloma are initially exposed to lenalidomide, usually in combination with bortezomib. Treatment options that are available for lenalidomide-refractory myeloma include pomalidomide- and carfilzomib-based +/- CD38 monoclonal antibody. The treatment options for daratumumab- and lenalidomide-refractory myeloma are increasing with pom-based treatments such as Pom cyclodex, elinexor + Velcade (bortezomib) + Dexamethasone (SVD), Melflufen, Taquita, and B-cell maturation antigen targeted therapeutic options. Therefore, when choosing a new treatment for patients with RRMM, it is crucial to use therapies with a new mechanism of action. Triplet combination should be preferred if possible and reserving the best options for later should be prevented. However, access and reimbursement challenges with novel treatments hinder this approach, leaving fewer options for second-line treatment for lenalidomide-refractory patients. The optimal treatment is easily available and balances efficacy, toxicity, and financial burden.

### Key learnings

• Patient journeys vary from frontline to first relapse setting; tailoring therapeutic strategies should be based on risk stratification

• Tailoring treatment in multiple myeloma should be based on patient needs and preferences, local regulatory and clinical policies and practices, treatment goals at each stage of the disease, and drug-related characteristics 

• Real-world evidence should guide changes in treatment guidelines and clinical practice to best support clinician decisions

• New treatment options give hope and offer improved quality of life; however, there is still no cure; thus, there needs to be flexibility in treatment goals with consideration given to tailored therapy options for newly diagnosed multiple myeloma and lenalidomide-sensitive patients

### Lymphoma

*Emerging role of positron emission tomography (PET) imaging and circulating tumor DNA (ctDNA)*

Research studies highlight the potential of baseline PET metrics and the integration of PET parameters into prognostic models and confirm that it is more sensitive than bone marrow biopsy in Hodgkin lymphoma (HL) and diffuse large B-cell lymphoma (DLBCL) [21-23]. Quantitative models based on positron emission tomography (PET) with computed tomography (PET-CT) have been shown to be superior to traditional clinical prognostic indices. PET-CT changes can potentially guide lymphoma staging and treatment modifications [24]. Such approaches can also facilitate the early recognition of refractory patients. Specific PET metrics need to be incorporated into routine lymphoma management, including establishing a consensus on measuring metabolic tumor volume (MTV), defining robust threshold values and prognostic indices for HL and non-Hodgkin lymphoma (NHL), and validating these indices in PET-based risk-adapted trials. However, the Lugano Classification does not support the use of PET for staging indolent lymphomas, such as marginal zone lymphoma (MZL), owing to their low 18F-FDG avidity [25]. 18-FDG PET-CT may be used to target biopsy in patients with suspected transformation. Given the superiority of PET quantitative-based models over traditional clinical prognostic indices, integrating the former into routine practice is necessary [25].

ctDNA plays a transformative role in lymphoma management and diagnostics, as lymphoma DNA is up to 1000-fold more abundant in plasma than peripheral blood mononuclear cells [26, 27]. In HL tissue samples, the proportion of tumor cells in masses is less, resulting in a genotyping that is not very informative [26, 27]. ctDNA can be used to determine copy number variations, tumor copy number abnormalities, and structural variance [28-30]. Baseline ctDNA in cerebrospinal fluid can be used to detect common mutations in central nervous system (CNS) lymphoma [31], capture genetic subtypes of DLBCL, and aid in the diagnosis of primary CNS lymphoma. Owing to the higher sensitivity and specificity, ctDNA may contribute to improved patient outcomes in lymphoma. Additionally, the false positive end-of-treatment PET-CT scans can be corrected using end-of-treatment MRD measured with ctDNA, providing clinicians with powerful tools to improve the accuracy of diagnosis, personalize treatments, and monitor response more effectively. ctDNA dynamics can also be used as potential early predictors of response in chimeric antigen receptor T cell (CAR-T)-treated patients [32].

### Recent therapeutic developments

Recent changes in the frontline and salvage regimen in relapsed/refractory Hodgkin lymphoma (RRHL) include the introduction of novel agents such as brentuximab vedotin and checkpoint inhibitors with a PET-adapted approach [33]. In chemo-refractory patients, real-world evidence suggests the use of brentuximab vedotin as a bridge to autologous stem cell transplantation (ASCT) [34]. Treatment of RRHL after ASCT failure includes targeted therapies, immunotherapy, second ASCT, allogeneic stem cell transplantation, conventional chemotherapy, and radiotherapy [35, 36]. PD-1 blockade before ASCT has also shown improved outcomes in RRHL. Additionally, allo-SCT and CD30 CAR-T therapies have improved survival in classical HL refractory or intolerant to brentuximab vedotin and anti-PD-1 therapy. PET interim has allowed clinicians to better tailor therapy as shown by RATHL, GHSG HD 18, and LYSA AHL2011 trials [37-39]. Nonetheless, the toxicity profile of first-line treatment has changed, with more hematological toxicity and peripheral neuropathy, less pulmonary toxicity, and autoimmune-related toxicity. There is a potential for bispecific antibodies to be included in front-line strategies. Frontline subcutaneous mosunetuzumab monotherapy and combination of mosunetuzumab with lenalidomide have shown promising results in MZL and follicular lymphoma (FL) in preliminary trials [40, 41]. 

CAR-T cell therapy represents a potentially curative treatment option for patients with relapsed refractory aggressive B cell lymphomas and has shown compelling results in patients with mantle cell and FL. Real-world data have confirmed positive outcomes for both aggressive histologies and FL [42, 43]. CAR-T therapies, specifically axi-cel and liso-cel, have been approved for the treatment of primary refractory disease or early relapse [44]. As per National Comprehensive Cancer Network (NCCN) recommendations, CAR-T can be used in second line for early relapse transplant-eligible patients [45, 46]. Understanding relapses after CAR-T therapy, affordability, and long-term effects remains a critical unmet medical need with CAR-T use. 

Recent evidence regarding the clinical and biological heterogeneity in T-cell lymphomas has highlighted the importance of targeting specific drive pathways in genetic subtypes of the disease and guiding new treatment approaches. Several trials are ongoing demonstrating the efficacy of agents targeting different pathways. As per the JACKPOT8 trial, golidocitinib has shown promising outcomes including anti-tumor efficacy across all peripheral T-cell lymphoma (PTCL) types [47]. Inhibition of enhancer of zeste homolog 2 (EZH2) using SHR2554 and inhibition of EZH1/2, enzymatic catalytic subunit of PRC2, using HH2853 have also shown promising outcomes in RR PTCL [48, 49] and are under further investigation. The REDIRECT study demonstrated that AFM13, targeting CD30+ lymphomas by enhancing the innate immune response, exhibited clinical efficacy in heavily-pretreated patients with PTCL.

### Key learnings

• Important advances in imaging and diagnostics are allowing for more precision in staging of lymphomas.

• Future use of ctDNA and PET-CeCT can allow for more personalized treatment approaches.

• CAR-T therapy represents a potential curative treatment strategy in some lymphomas.

• For T-cell lymphomas, it is necessary to target specific pathways in the genetic subtypes of the disease to overcome the clinical and biological heterogeneity, which is the focus of several ongoing trials.

### Lung cancer

*Molecular profiling*

Genomic-driven cancer medicine is utilized throughout the patient’s journey, including diagnosis, determining clinical response, and identifying new therapies in cases of drug resistance [50]. Several molecular profiling tools are now available for NSCLC, such as FISH, polymerase chain reaction (PCR), next-generation sequencing (NGS), and immunohistochemistry (IHC) [50]. NGS is the backbone of the genomics era in cancer medicine as it performs massive parallel sequencing, thereby overcoming the limitations of single biomarker testing. This reduces the turnaround time and makes it more cost-effective [51, 52]. The Asia-Pacific Drug Development Consortium Working Group recommends a focused panel multiplex-gene NGS for NSCLC to save cost and time [53]. The approach to NGS use in lung cancer in routine evaluation can be tailored to suit the relevant population. It is currently possible to detect up to 45% of actionable genetic mutations involved in NSCLC. In Asian populations, 22% of genetic mutations are not actionable. Epidermal growth factor receptor (EGFR) mutations are the most common oncogenic drivers in NSCLC and are most prevalent in Asians [54-58]. Real-world evidence suggests that 49.1% and 51.4% of the exon20ins missed by PCR in the GENIE trial and FoundationInsights trial, respectively, were detected by NGS, highlighting the underdiagnosis of EGFR exon20ins mutation variants with PCR [59].

### Optimizing clinical management with targeted treatment

Treatment options in the early stages of NSCLC are rapidly evolving in the adjuvant, neoadjuvant, and perioperative settings. Currently, targeted therapy and immunotherapy are being employed in adjuvant, neoadjuvant, and perioperative settings. Trials focusing on adjuvant EGFR tyrosine kinase inhibitor (TKI) and anaplastic lymphoma kinase (ALK) inhibitors, such as ADJUVANT/CTONG 1104, ADAURA, and ALINA, have demonstrated positive outcomes [60-62]. For advanced ALK+ lung cancer, European Society of Medical Oncology (ESMO) and NCCN guidelines recommend all three generations of ALK inhibitors such as alectinib, brigatinib and lorlatinib, noting significant improvement in PFS with second- and third-generation agents [63, 64]. Patients with ALK+ NSCLC often have brain metastases; thus, American Society of Clinical Oncology and ESMO guidelines recommend TKIs effective in the brain and suggest avoiding local therapy initially owing to the strong brain activity of ALK inhibitors [63, 64]. The toxicity of these agents is manageable. Ongoing trials indicate promising results for ALK TKIs in neoadjuvant settings. The use of NGS, ctDNA analysis, and rebiopsies is recommended to personalize treatment with TKIs and prevent resistance [65, 66]. Targeted therapy has also shown promise for other mutations apart from EGFR, such as RET fusion (selpercatinib or pralsetinib) and MET exon 14 skipping mutations (capmatinib or tepotinib). Immunotherapy offers greater advantages in the neoadjuvant settings than in adjuvant settings [67-71]. Antibody drug conjugates have shown promising results for *HER2* mutations and are approved in later line settings. Additionally, novel immune checkpoint targets beyond *PD-L1* have been identified, such as LAG3, TIM3, and TIGIT [72]. These checkpoints appear complementary to currently targeted checkpoints and combined inhibition could augment or improve the clinical outcomes. Nevertheless, these checkpoints should not be used as monotherapy as the effect of PD-1 blockade is proportionally larger than that of LAG-3, TIM-3, or TIGIT blockade alone [73].

### Key points

• Molecular profiling and NGS are paving the way for a more tailored approach to disease management.

• A multidisciplinary team approach is needed to integrate recent advances into clinical practice and support a more personalized approach to therapy.

• Next generation immunotherapies are urgently needed for more improvement in patients with NSCLC without oncogenic driver. 

### Current challenges with cancer care in APAC

One of the major challenges faced in the APAC region is the limited access to novel treatments and the lack of insurance and reimbursement, making these therapies unaffordable. The availability of drugs and limited experience with novel agents such as CAR-T therapies are also major issues, limiting the options available for patient care. Additionally, the understanding of the different treatments and their role is limited among health care practitioners in this region to allow tailored treatments. 

Regarding recent advances in diagnostic approaches, obtaining good samples and storing and handling them for liquid biopsies is a challenge that limits the quality of the liquid biopsy test results. The handling of liquid biopsy samples obtained from solid cancer should involve proper tubes for collection and shipment to ensure that the cell-free DNA does not degrade. Additionally, several other practical limitations exist with the use of NGS and liquid biopsies, such as cost and reimbursement, tissue availability, time sensitivity, and challenges in data handling and interpretation, that need to be considered [74]. The availability and experience with newer technologies is also lacking.

### Future insights

With the rise in cancer burden in the APAC region, there is a need to focus on early detection, approvals of treatment options, and personalized treatment. Interim PET scans and ctDNA analysis are anticipated to inform tailored treatments in solid cancers in future. With the diverse number of novel treatment strategies available, understanding of the molecular heterogeneity and immune system is essential for the rational use of novel treatments and tackle the heterogeneity observed within certain cancers. A combination of various drugs and newer technology for detection and staging is required for personalized treatment and improving the quality of life. Given the heterogeneity seen within different populations in the APAC region, the need for precision medicine, newer technology for detection and staging, tailored treatment using a combination of drugs, and toxicity management are imperative. Future strategies to improve cancer care, especially in the APAC region, should explore initiatives of including newer technologies, such as total metabolic tumor volume and ctDNA, into staging and/or prognostic systems, incorporating MRD evaluation in select histologies, and developing common staging and restaging criteria that are suitable for the most common histologic subtypes.

### Conclusions

The Onco Summit 2024 : The APAC Chapter successfully emphasized the prevalent and ever-rising challenges in oncology and facilitated the exchange of global best practices. By emphasizing recent advances, especially immunotherapy and targeted therapies, the Summit sheds light on overcoming common challenges in delivering optimal cancer care in the APAC region. An emphasis was laid on the variety of treatment options that are not available owing to lack of financial support and approvals. Additionally, the role of newer technologies such as NGS and liquid biopsies in precision medicine was highlighted, which need to be made available in all countries in the APAC region. Through the unbiased discussions around adaptable strategies and collaborative approaches, the Summit proved pivotal for a region grappling with significant challenges and obstacles to cancer care. More importantly, the Summit established a network of oncologists, hematologists, and scientists in the APAC, for advancing global and regional cancer care, and provided an opportunity for the HCPs to create new collaborations and discuss future research projects. 

## Author Contribution Statement

All authors were involved in conceptualization, formal analysis and verification of all data, interpretation of information, and writing and reviewing of all drafts of this manuscript. All authors had access to and verified all the data reported in this manuscript and accept responsibility for this publication. All authors have reviewed and approved this manuscript for publication. 
